# Kartogenin (KGN)/synthetic melanin nanoparticles (SMNP) loaded theranostic hydrogel scaffold system for multiparametric magnetic resonance imaging guided cartilage regeneration

**DOI:** 10.1002/btm2.10364

**Published:** 2022-06-29

**Authors:** Chuyao Chen, Shaoshan Huang, Zelong Chen, Qin Liu, Yu Cai, Yingjie Mei, Yikai Xu, Rui Guo, Chenggong Yan

**Affiliations:** ^1^ Department of Medical Imaging Center, Nanfang Hospital Southern Medical University Guangzhou China; ^2^ Key Laboratory of Biomaterials of Guangdong Higher Education Institutes, Guangdong Provincial Engineering and Technological Research Centre for Drug Carrier Development, Department of Biomedical Engineering Jinan University Guangzhou China; ^3^ Clinical Research Center Zhujiang Hospital, Southern Medical University Guangzhou Guangdong China; ^4^ Center of Orthopedics, Zhujiang Hospital Southern Medical University Guangzhou Guangdong China; ^5^ School of Biomedical Engineering Southern Medical University Guangzhou China

**Keywords:** cartilage regeneration, hydrogel, kartogenin, magnetic resonance imaging (MRI), theranostics

## Abstract

Cartilage regeneration after injury is still a great challenge in clinics, which suffers from its avascularity and poor proliferative ability. Herein we designed a novel biocompatible cellulose nanocrystal/GelMA (gelatin‐methacrylate anhydride)/HAMA (hyaluronic acid‐methacrylate anhydride)‐blended hydrogel scaffold system, loaded with synthetic melanin nanoparticles (SMNP) and a bioactive drug kartogenin (KGN) for theranostic purpose. We found that the SMNP‐KGN/Gel showed favorable mechanical property, thermal stability, and distinct magnetic resonance imaging (MRI) contrast enhancement. Meanwhile, the sustained release of KGN could recruit bone‐derived mesenchymal stem cells to proliferate and differentiate into chondrocytes, which promoted cartilage regeneration in vitro and in vivo. The hydrogel degradation and cartilage restoration were simultaneously monitored by multiparametric MRI for 12 weeks, and further confirmed by histological analysis. Together, these results validated the multifunctional hydrogel as a promising tissue engineering platform for noninvasive imaging‐guided precision therapy in cartilage regenerative medicine.

## 1 INTRODUCTION

Articular cartilage injury and pathological changes caused by trauma or chronic diseases eventually lead to joint pain and functional disability, which is a common clinical disease.^1^ Due to its avascularity, low cellularity, and poor migration ability, it is difficult for cartilage tissue to gain self‐repair after injury.^2^ Traditional surgical procedures for articular cartilage injuries include debridement, bone marrow stimulation, autogenous osteochondral transplantation, allogeneic osteochondral transplantation, chondrocyte implantation, and joint replacement.^3^ However, the efficacy of these methods remains unsatisfactory in the clinical treatment of minor articular cartilage symptoms. Debridement and bone marrow stimulation techniques (such as microfractures), as the first‐line treatment, are prone to the formation of fibrocartilage that cannot withstand the articular stress over time.^4^ Other widely used techniques like osteochondral grafts or chondrocyte implantation are limited by donor site morbidity, inadequate donor supply, graft failure, and stratification.^5^

Recently, tissue engineering strategies containing combinations of stem cells, biomaterials (scaffolds) and/or functional biomolecules have raised interest in the field of articular cartilage repairment.^6^ Bioactive factors are widely used to promote the differentiation of bone marrow mesenchymal stem cells (BMSCs) to chondrocytes.^7^ Particularly, kartogenin (KGN) is a small non‐protein compound that induces mesenchymal stem cells to homing.^3^ Some attempts have been conducted to promote chondrocyte differentiation effectively by KGN in the treatment of osteoarthritis.^5^, ^8^, ^9^ However, injecting KGN into the articular cavity directly will face problems like loss of KGN or absorption into the circulatory system.^10^ Consequently, a continuous‐release system is needed to sustainably prolong the activity of KGN to repair the damaged cartilage. More recently, Xu et al.^11^ prepared biocompatible and degradable scaffold loaded with KGN and BMSCs, which facilitated a permanent release of small growth factors and enhanced chondrogenesis of encapsulated BMSCs. In addition, supramolecular injectable hydrogel containing stem cells can effectively promote hyaline cartilage and subchondral bone regeneration in a rat model of cartilage defect.^12^

As is known to all, it is important to find noninvasive methods to monitor the functionalization and degradation of tissue engineering scaffolds longitudinally in vivo. Onofrillo et al^13^ recently designed a fluorescently labeled sensitive hydrogel to correlate the degradation of a scaffold with cartilage formation. Moreover, Xiao and colleagues^14^ developed a cationic photoacoustic contrast agent to image cartilage degeneration. However, the further application of fluorescence imaging and photoacoustic imaging were limited by their poor resolution.

Magnetic resonance imaging (MRI) has been widely used to assess the morphological changes of biomaterial degradation and new tissue reconstruction for its safety and noninvasive measurement.^15^ It has obvious advantages in the evaluation of various regeneration strategies with ideal soft‐tissue resolution and penetration depth.^16^ For example, Hong et al^17^ reported a chitosan modified Fe_3_O_4_‐CS/KGN nanoprobe for osteochondral diagnosis and repair. The Fe_3_O_4_‐CS/KGN nanoprobe can act as a diagnostic tool for defect position distinguishment and dynamic observation. We previously designed a theranostic scaffold system fabricated from ultrasmall superparamagnetic iron oxide‐labeled hydrogel. The multifunctional scaffold showed favorable MRI contrast and mechanical properties, which allowed the noninvasive monitor and analysis of both hydrogel degradation and cartilage repair in situ.^18^ Compared with traditional iron oxide nanoparticles, synthetic melanin nanoparticles (SMNP) have excellent biocompatibility and ability to coordinate separation of paramagnetic metal centers.^19^ The functional catechol network of SMNP facilitates the scaffold for paramagnetic metal ion chelation to be applied to a *T*_1_ weighted MRI contrast agent.^20^

**FIGURE 1:**
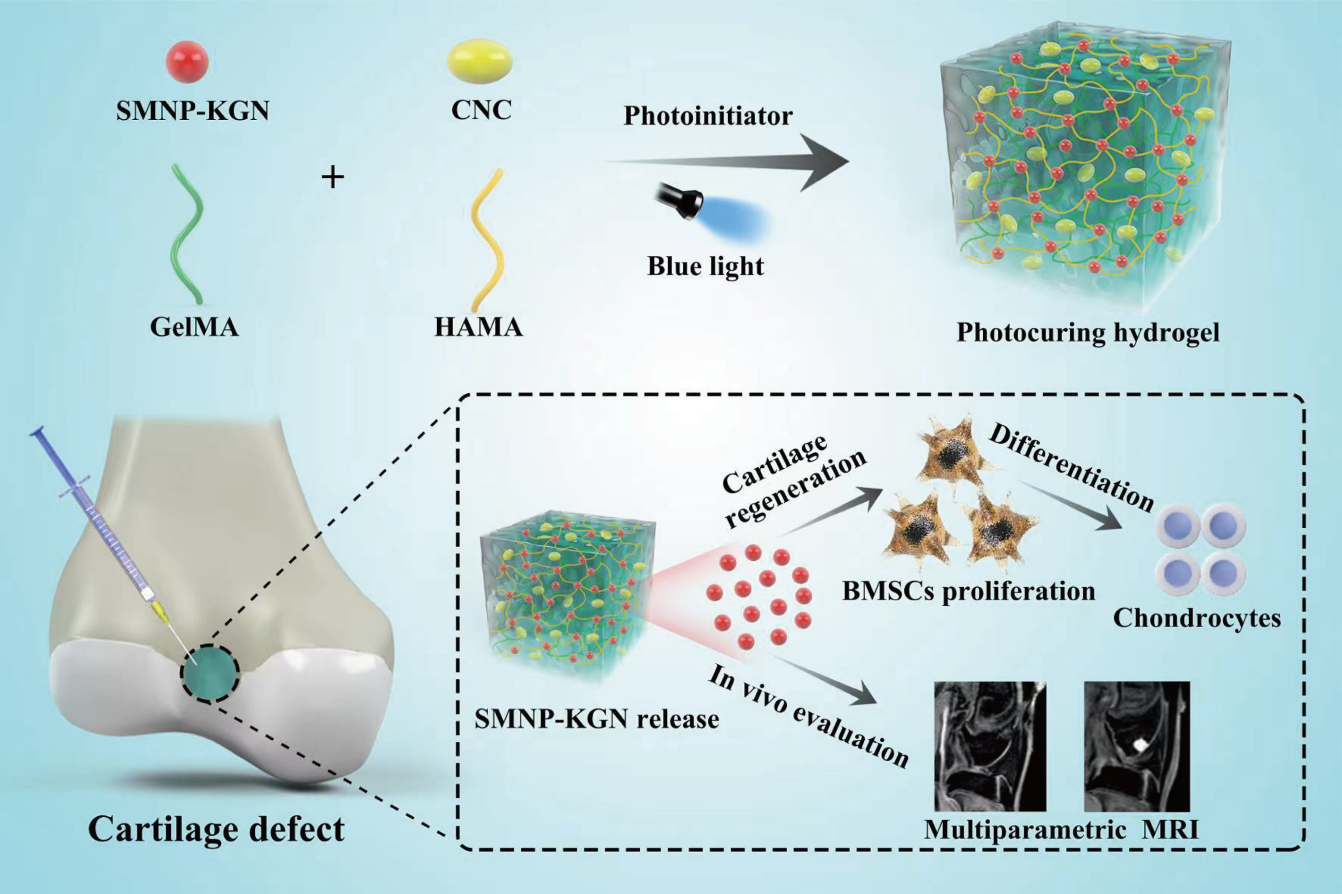
Schematic illustration of preparation and functionalization of kartogenin loaded and SMNP labeled multifunctional hydrogel scaffold for cartilage regeneration

Here, we synthesized an injectable GelMA/HAMA/CNC hydrogel scaffold incorporated with KGN and SMNP (Figure 1). The biofunctional hydrogel scaffold system can provide a microenvironment matrix to support sustained release of KGN, which promotes the homing of BMSCs and induces chondrogenic differentiation. Moreover, the cartilage regeneration and SMNP‐KGN/Gel degradation were evaluated by quantitative MRI analysis longitudinally in a rabbit articular cartilage defect model. The in vivo imaging results were further confirmed by histological and pathological analysis.

## 2 RESULTS

### 2.1 Characterization of the hydrogel

**FIGURE 2:**
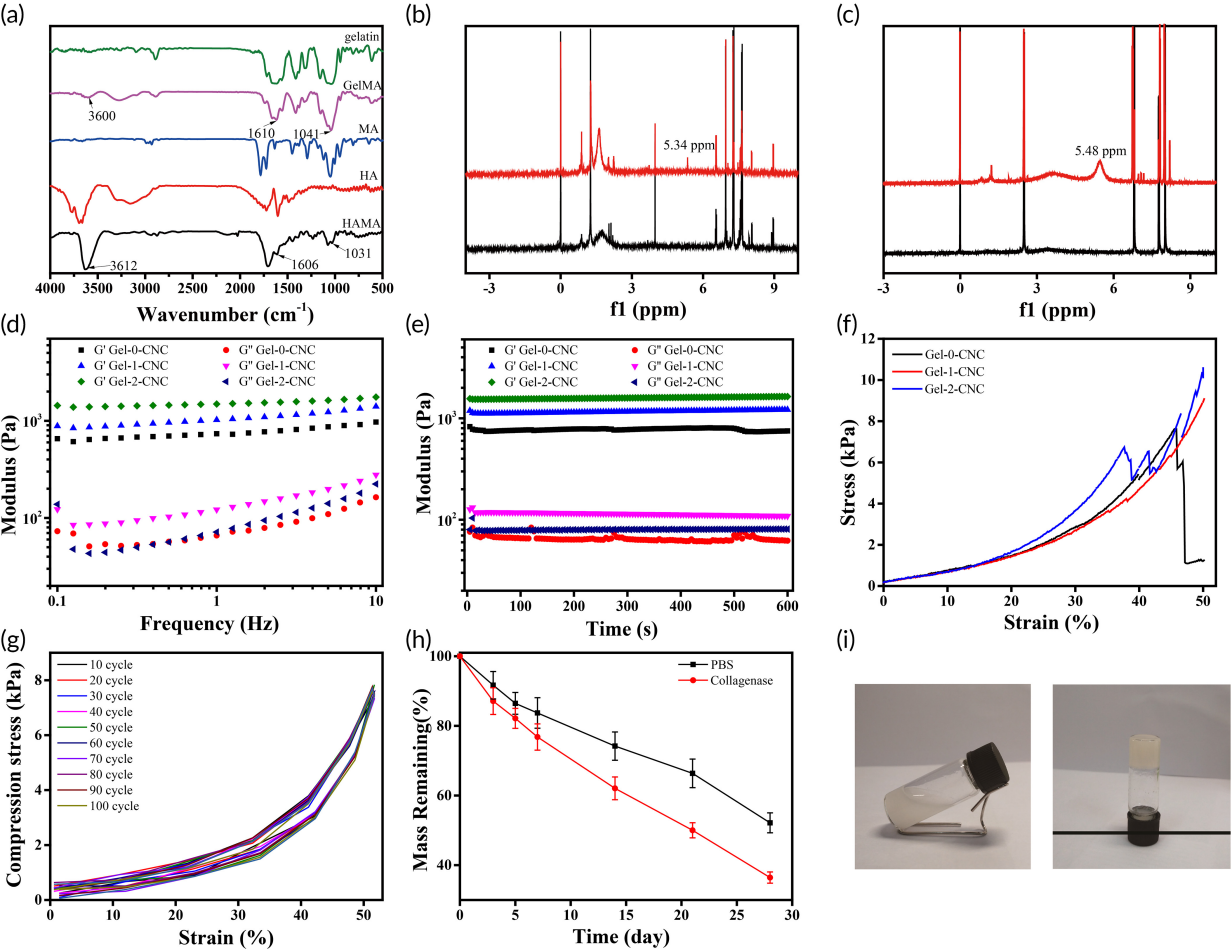
(a) FTIR of materials of hydrogel. (b) ^1^H NMR spectra of gelatin, GelMA, (c) hyaluronic acid and HAMA. (d) Modulus over frequency. (e) Modulus over time. (f) Stress–strain curve of Gel‐0‐CNC, Gel‐1‐CNC, and Gel‐2‐CNC hydrogels under static compression. (g) Stress–strain curve of Gel‐1‐CNC hydrogel under cyclic compression over 100 cycles. (h) In vitro degradation analysis of hydrogels immersed in PBS solution and collagenase at 37°C. (i) The hydrogel gelation process. (*n* = 3, mean ± SD)

The FTIR spectrum (Figure 2a) showed the successful synthesis of both GelMA and HAMA. FTIR spectra showed an absorption peak of twisting vibration attributable to a CH_2_ group at 1029 cm^−1^, and absorption peak at 1610 cm^−1^ attributed to stretching vibration of C=C group, indicating a presence of methacryl groups in the chemical structure of GelMA.^21^ Besides, the absorption peak at 1031 and 1606 cm^−1^ also appeared in the spectra of HAMA. Furthermore, ^1^H NMR of the GelMA (Figure 2b) and HAMA (Figure 2c) exhibited the methacrylamide vinyl group signal that increased at 6.0 ppm and 5.0 ppm, indicating MA modified the gelatin and hyaluronic acid successfully.^22^ From Figure S1, the pore size distribution of the Gel‐0‐CNC, Gel‐1‐CNC and Gel‐2‐CNC hydrogel was 327 ± 80.37 μm, 234 ± 60.66 μm and 174 ± 60.25 μm, respectively. The prepared hydrogel was suitable for cell metabolism and cartilage regeneration.^23^

The effect of different composition on the rheological properties of the hydrogel was explored by the storage modulus (*G*′) and loss modulus (*G*″). All hydrogels could maintain a gel state with the time (Figure 2d) and frequency (Figure 2e) increased. However, the Gel‐1‐CNC and Gel‐2‐CNC hydrogel presented higher storage modulus (G′) than the Gel‐0‐CNC hydrogel, which can be attributed to the formation of percolated network of CNC particles within hydrogel.^23^ The mechanical properties of the hydrogels with different contents of CNC were measured. From the Figure 2f, the maximum stress of the Gel‐0‐CNC, Gel‐1‐CNC, and Gel‐2‐CNC were 7.63 kPa, 9.03 kPa and 10.57 kPa, respectively. From the results that CNC can indeed enhance the mechanical properties of the hydrogel. But as the CNC concentration increases, the mechanical properties of hydrogels change indistinctively.

From this, the Gel‐1‐CNC hydrogel was chosen to explore the subsequent studies. Then, the Gel‐1‐CNC was subjected to 100 cycles of loading/unloading by applying a 50% strain (Figure 2g). The hydrogels produced a stress–strain curve like the original hydrogels, proving that they exhibited good anti‐fatigue performance. Moreover, suitable degradation rate of hydrogels is also a significant consideration for biomaterials.^24^ In this study, we explored the degradation rates of Gel‐1‐CNC hydrogels in PBS and collagenase solution (Figure 2h and Figure S2). The mass degradation rate of Gel‐1‐CNC was 47.8% in the in PBS and 53.6% in the hyaluronidase solution after 28 days at pH = 7.4. The Gel‐1‐CNC hydrogels showed higher degradation rate in collagenase, mainly because the main chain of gelatin is destroyed.^25^ But in the PBS, the degradation is mainly based on the hydrolytic scission of molecular chains, and broken of the amide bond and polysaccharide‐ride chain. The hydrogels exhibited similar degradation properties under different pH conditions. The mass degradation rate of Gel‐1‐CNC was 35.3% in the in PBS and 51. 2% in the hyaluronidase solution after 28 days at pH = 5.5. From the Figure 2i, after the irradiation of blue light, the hydrogel was quickly formed. To conclude, the hydrogels exhibited suitable pores, excellent stability, and good mechanical properties, revealing their potential application in cartilage regeneration.

### 2.2 Characterization of SMNP and SMNP‐KGN nanoparticle

**FIGURE 3:**
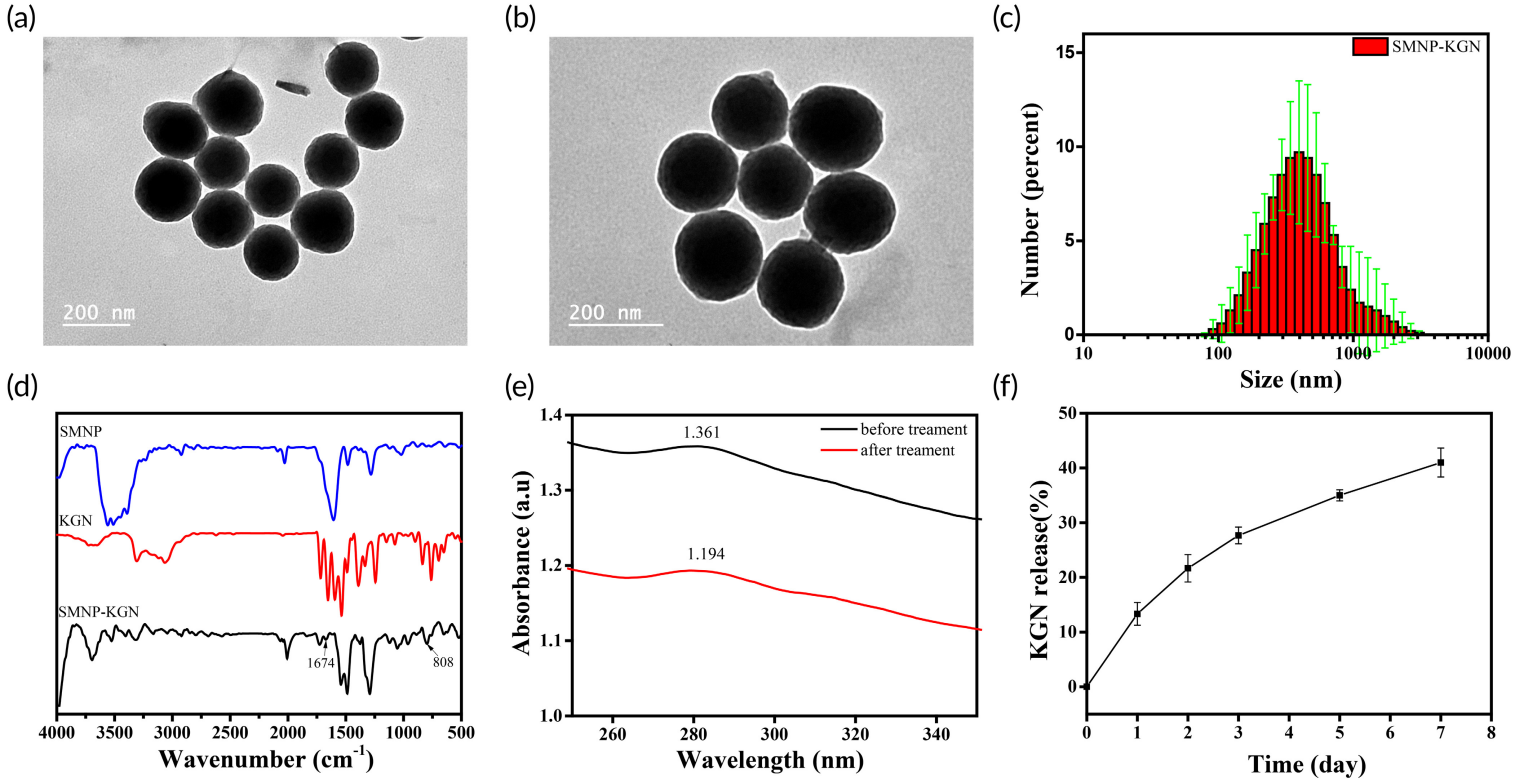
(a,b) TEM of SMNP and SMNP‐KGN. (c) The size distribution of SMNP‐KGN measured by DLS. (d) FTIR spectra of different components. (e) Ultraviolet spectrophotometer detection of KGN conjugated to SMNP. (f) KGN release of SMNP‐KGN/Gel hydrogels in the PBS. (*n* = 3, mean ± SD)

The SMNP with natural function and structure maintain lots of ideal properties of natural melanin. SMNP have tremendous potential in its utility across an extensive range of biomedical applications.^26^ The SMNP and SMNP‐KGN surface morphologies were observed by TEM. The SMNP had a diameter of 188 ± 19.76 nm (Figure 3a), while the hydrodynamic size measured by DLS was 200–300 nm (Figure S3). The KGN‐grafted SMNP nanoparticles (SMNP‐KGN) also displayed spherical shapes (Figure 3b), the hydrodynamic size was 300 nm (Figure 3c), due to the KGN molecules grafted with the SMNP nanoparticles. The FTIR spectrum further proved that SMNP‐KGN had been successfully synthesized. From Figure 3d, the characteristic peak at 1670 cm^−1^ that presented in SMNP‐KGN was by reason of the hydrazone bonds formed between SMNP and KGN via amide reaction.^27^ The characteristic peak at 647 cm^−1^, which was attributed to Fe‐O stretching vibrations, confirming that KGN was grafted on the SMNP.

SMNP‐KGN conjugated ability was evaluated by ultraviolet–visible spectrophotometry. From the Figure 3e, the maximum absorption peak before and after adding SMNP was 1.36 and 1.19, respectively. We calculated that the conjugated KGN contributed approximately 12.27% to the absorption. We further assessed the KGN release behavior of KGN‐loaded hydrogels (Figure 3f). KGN could be released through the hydrogel barrier in the environmental liquid by breaking from the amide bond of SMNP‐KGN under the action of water molecules.^28^ According to the standard curve Figure S4, the KGN released from the SMNP‐KGN/Gel reached 41% after 7 days. As shown in Figure S5, the SMNP‐KGN incorporated hydrogels were scanned by *T*_1_‐weighted MR imaging in vitro. With the increase of Fe concentration, the contrast effect of hydrogels on *T*_1_‐weighted MRI was significantly enhanced. To study the *T*_1_ relaxation (*r*_1_), the Fe concentration in SMNP‐KGN was used to fit the curve (1/*T*_1_ relative to Fe concentration). The *r*_1_ value was calculated as 7.95 mm^−1^ s^−1^ according to the corresponding fitting line slope.

### 2.3 The biocompatibility and bioactivity of SMNP‐KGN/Gel in vitro

**FIGURE 4:**
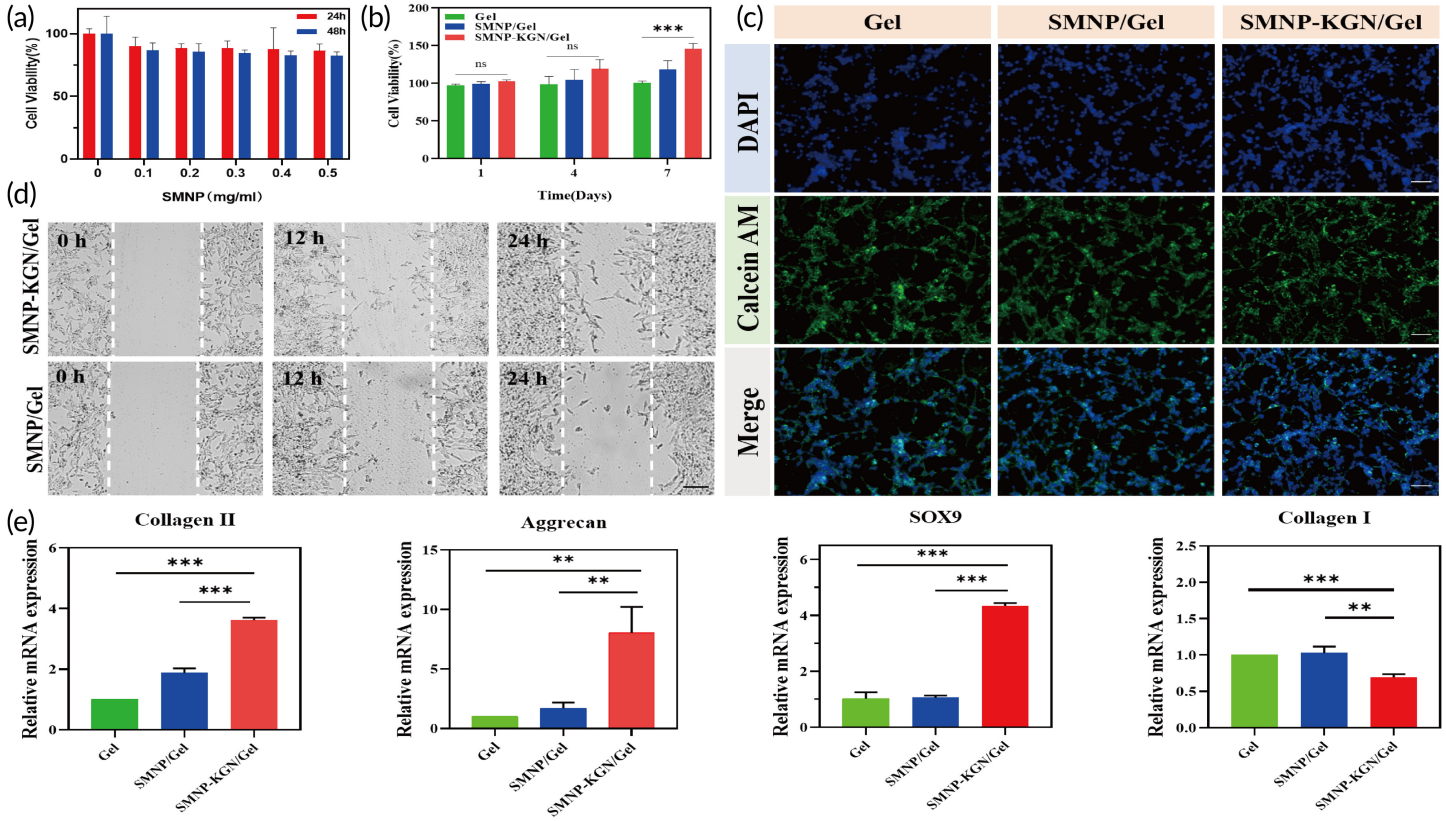
The biocompatibility and bioactivity of SMNP‐KGN/Gel in vitro. (a) Cytotoxicity experiments of hydrogels with increasing concentrations of SMNP in 24 h and 48 h. (b) Cell proliferation experiments after incubation for 1, 4, and 7 days. (c) Live/dead assay observed under the fluorescence microscope on day 7. Scale bar: 100 μm. (d) Cell migration evaluated by the cell scratch wound experiment. Scale bar: 200 μm. (e) COLII, Aggrecan, SOX9, and COLI expression levels during chondrogenesis in vitro

To verify the biocompatibility of the SMNP labeled hydrogels in vitro, the cell viability was measured by the CCK‐8 assay. The cell viability at the highest SMNP concentration (0.5 mg/mL) at 24 h and 48 h was 86.42% and 82.34%, respectively (Figure 4a), which demonstrated low cytotoxicity of the SMNP. We also investigated the BMSCs proliferation on SMNP‐KGN/Gel. As shown in Figure 4b, no significant difference was found in the cell viability between day 1 and day 4. However, the cell viability in the SMNP‐KGN/Gel group on day 7 was significantly higher than that in the control group (146.00% versus 102.62%, *P* < 0.05), suggesting that SMNP‐KGN/Gel provides a favorable environment for the growth of BMSCs. Calcitonin AM/PI staining (Figure 4c) showed that PI‐labeled cells were rarely captured, indicating low cytotoxicity of the as‐prepared hydrogel. Interestingly, on day 7, BMSCs were flat and fusiform, and aggregated into condensed clusters for chondrogenic differentiation.^29^ In the cell migration experiment, BMSCs stimulated by KGN migrated faster in the wound healing model (Figure 4d), which suggested that the KGN‐loaded hydrogels can facilitate host BMSCs homing to enhance hydrogel‐host tissue integration.^30^ These cellular experiments demonstrated that the SMNP‐KGN/Gel had good biocompatibility and could promote the proliferation and migration of BMSCs in vitro.^31^, ^32^

The expression levels of chondrogenic markers genes (aggrecan, Sox9, and collagen II) and a dedifferentiation‐related gene (collagen I) in the hydrogels were evaluated. As shown in Figure 4e, the expression of aggrecan, Sox9, and collagen II were significantly enhanced in the SMNP‐KGN/Gel after 14 days. This can be attributed to the fact that KGN activates protein transcription, such as type II collagen and aggrecan, involved in cartilage matrix components.^33^ In contrast, the expression of collagen I remained in low value (relative mRNA expression = 0.69) in the SMNP‐KGN/Gel, which indicated that the KGN‐loaded hydrogel promotes BMSCs for chondrogenic differentiation instead of the fibrocartilage phenotype.^34^ These results indicated that the SMNP‐KGN hydrogel could enhance BMSCs chondrogenesis in vitro.

### 2.4 MRI and macroscopic evaluation in vivo

**FIGURE 5:**
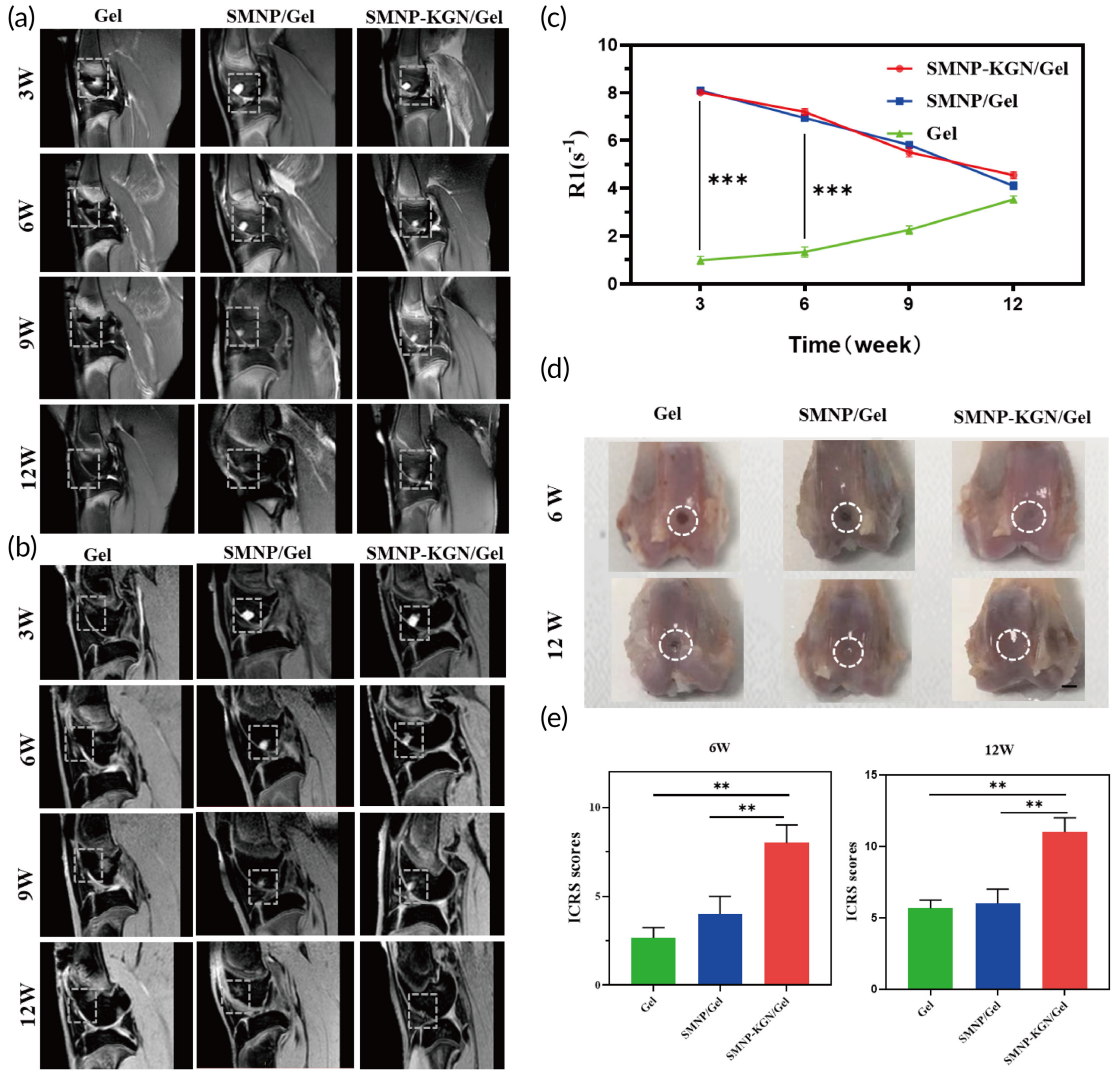
MRI and macroscopic analysis of cartilage defect model. (a) MR proton‐density‐weighted imaging (PDWI) for cartilage evaluation at 3, 6, 9, and 12 weeks after surgery. (b) MR water‐selective cartilage scan (3D_WATSc) for the morphological observation of the degradation of hydrogel. (c) Semiquantitative *R*_1_ relaxometry rate comparison between different construction of hydrogels. (d) Observations of harvested cartilage at week 6 and 12 after surgery (white circle: degenerated cartilage). The scale bar indicates 5 mm. (e) ICRS scores of PBS group, SMNP/Gel and SMNP‐KGN/Gel at week 6 and 12 post‐surgery, respectively

To investigate the efficacy and degradation of SMNP‐KGN/Gel in vivo, multiparametric MRI was performed. At week 3, 6, 9, and 12 after the surgery, multiple MR sequences were scanned. The neocartilage was elevated with PDWI and 3D‐WATSc longitudinally (Figure 5a,b). In the SMNP labeled groups, the defect boundary could be clearly observed at 3 and 6 weeks, which can be attributed to the lengthwise proton relaxation effect of the loaded SMNP.^20^ The MRI enhanced signal of the SMNP labeled hydrogels gradually decreased within 12 weeks due to the release and absorption of SMNP from hydrogels. At week 12, smooth and continuous articular cartilage could be seen in the SMNP‐KGN/Gel group on 3D‐WATSC images. This may be due to KGN simulated chondrogenesis.^35^ In contrast, discontinuous cartilage surface with similar regeneration efficiency were observed in the Gel and SMNP/Gel groups. Moreover, as shown in Figure 5c, *R*_1_ values in the SMNP/Gel group (from 8.10 s^−1^ at week 3 to 4.10 s^−1^ at week 12) and the SMNP‐KGN/Gel group (from 8.02 s^−1^ at week 3 to 4.55 s^−1^ at week 12) gradually decreased over time, while *R*_1_ values in the Gel group remained low (0.99–3.54 s^−1^). Notably, *R*_1_ values of the SMNP/Gel group and the SMNP‐KGN/Gel group were significantly higher than those of Gel group at week 3 and 6 (both *P* < 0.001). These findings indicate that SMNP can be served as effective MRI contrast agents in vivo.

At week 6 and 12, the samples of femoral condyles were harvested and photographed (Figure 5d). After 6 weeks of treatment, the SMNP‐KGN/Gel group showed the most significant cartilage regeneration, and the defect was mostly recovered with thick and irregular neocartilage. However, no marginal tissue repair was observed in the groups without KGN. Meanwhile, hydrogel remnants were still visible in all the three groups at week 6 and were almost absorbed finally. After 12 weeks of treatment, regenerated cartilage‐like tissue was observed in the SMNP‐KGN/Gel group, and the defects were restored with smooth surfaces without ambient cartilage degeneration. In comparison, the two groups without KGN showed incomplete repair with irregular central cracking. The International Cartilage Repair Society (ICRS) macroscopic scores were used for regeneration evaluation.^36^ The osteochondral regeneration was evaluated in the aspect of total score, structural characteristics, degree of integration, joint surface regularity, safranin‐O staining, and subchondral morphology. As shown in Figure 5e, the score of the KGN‐treated group was significantly higher than that of other groups in week 6 and 12, which correlated well with the changes of MRI signal.

### 2.5 Histological evaluation in vivo

**FIGURE 6:**
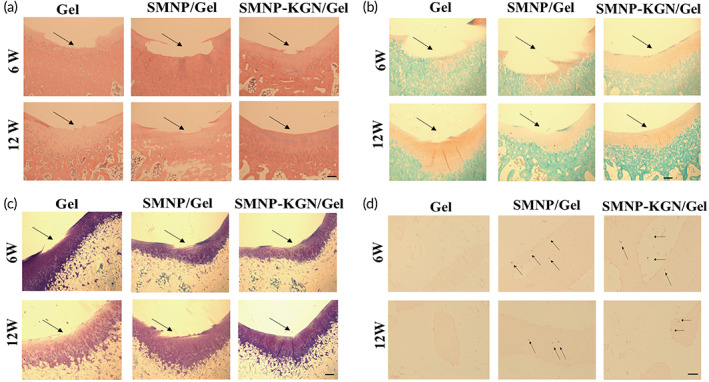
Histological evaluation at week 6 and 12 after surgery. (a) H&E staining for cartilage regeneration and hydrogel degradation assessment. (b) Safranin‐O staining and (c) Toluidine blue staining for the deposition of neocartilage (black arrows: the margins of the defection). (d) Prussian blue staining for the distribution of SMNP in cartilage tissues (black arrows: SMNP). Scale bars (a–c) indicate 200 μm, (d) indicate 100 μm

The process of biomaterial absorption and cartilage regeneration was confirmed by histological evaluation. At week 6, H&E showed small amounts of hydrogels remnants in all groups (Figure 6a). After that, differentiated chondrocytes were observed in SMNP‐KGN/Gel treated group. At week 12, the hydrogels were almost absorbed. The H&E staining demonstrated the smooth superficial layer with the surrounding extracellular matrix in the KGN‐treated group. For the SMNP‐KGN/Gel group at week 12, Safranin‐O staining (Figure 6b) and Toluidine blue staining (Figure 6c) showed more uniform proteoglycan accumulation and neocartilage matrix‐forming, indicating new and intact cartilage formation. In contrast, the two groups without KGN showed slight repair signs. The cartilage defect was covered by regenerated tissue, and the tidal scale and surface fiber structure were incompletely formed. Moreover, intense iron staining was shown in the residual SMNP labeled hydrogel scaffolds by Prussian blue staining (Figure 6d) at week 6, contributing to the preserved MRI relaxation rate (Figure 5).

## 3 DISCUSSION

With the development of cartilage tissue engineering, hydrogels containing growth factors are widely used for cartilage repair.^37^ Hydrogels with three‐dimensional expansion network facilitate mesenchymal stem cells migration and provide an ideal microenvironment for their proliferation and differentiation.^30^ In this study, we developed an injectable GelMA/HAMA/CNC hydrogel formed by lights, which was highly biocompatible and flexible to various shapes of defects for in vivo applications.

The ideal tissue engineering scaffold should be biodegradable to facilitate the regenerated tissue completely replaced by the graft. Previous studies have proved that HA is an ideal choice for the manufacture of cartilage regeneration engineering materials, because its degradation and drug release can adapt to the biochemical process in vivo.^38^ Our H&E staining results also demonstrated that GelMA/ HAMA/CNC hydrogels could be completely degraded and absorbed in animal model. Normally, the mechanical strength of hydrogel is not as high as that of normal cartilage.^30^ The enhancement effect of CNC fibers on the mechanical properties of polymers has been reported previously.^39^ In this study, we found that 1% of CNC addition increased the mechanical strength of hydrogels and maintained the three‐dimensional network structure of water swelling polymers stable.

KGN, a small molecule drug promoting cartilage formation first reported in 2012,^8^ can effectively induce hMSCs to differentiate into chondrocytes. Some studies have shown that KGN is stable at room temperature and has good biocompatibility with no significant toxicity to normal cells.^40^ In consistent with previous studies,^2^, ^30^, ^41^, ^42^, ^43^ we demonstrated that KGN could effectively recruit extracellular BMSCs, promote their proliferation, and induce them to differentiate into cartilage. Kang et al used KGN as a cartilage repair accelerator by intraarticular injection in a rabbit osteoarthritis cartilage defect model, and found that KGN could inhibit cartilage degeneration and improve the repair quality of full‐thickness cartilage defect.^44^ In this study, a KGN loaded hydrogel was designed to support in situ cartilage regeneration. The ICRS score was significantly different between the KGN group and non‐KGN group. All of these results suggest that KGN is expected to be an ideal drug to promote cartilage repair.

Although KGN loaded hydrogels can effectively promote cartilage regeneration, the real‐time and noninvasive visualization of its in‐situ fate is important. Patients may require repetitive interventions if the implants degrade too fast. As we know, X‐ray is a popular choice for follow‐up clinically. However, it failed to track the fate and function of the implanted materials for its poor spatial resolution.^45^ Nowadays, effective methods such as fluorescence imaging^13^ and photoacoustic imaging^14^ to monitor the grafts or cartilage status have been studied. But they were unable to evaluate the implants and chondrogenesis simultaneously. MRI is an appropriate imaging tool to monitor the structural and biochemical changes of articular cartilage.^46^ Cartilage thickness, *T*_2_ and *T*_1q_ relaxation time have been proved to be potential imaging markers, which can provide insight into the degree of cartilage injury and dysfunction.^47^, ^48^ Meanwhile, multiple MRI sequences facilitate the evaluation of cartilage regeneration and grafts degradation simutaneously. Plenty of studies focus on different functional MRI sequences on cartilage regeneration, such as *T*_2_ mapping, *T*_2*_ mapping, *T*_1ρ_, delayed gadolinium enhanced MRI of cartilage, sodium imaging, and glycosaminoglycan chemical exchange saturation transfer. So far, *T*_1ρ_ mapping and delayed gadolinium enhanced MRI are promising methods in cartilage evaluation.^9^ Sodium imaging and gagCEST are preclinical technologies, which depend on high field strength and complex software and hardware.^49^

In this study, we grafted KGN onto the synthesized SMNP, which showed higher *r*_1_ than other iron‐based contrast agents reported in the literature.^50^, ^51^, ^52^ Our in vivo MRI results showed that the incorporation of SMNP‐KGN made the hydrogel in the defect area clearly observed on the MR images, and the changes of relaxation rate *R*_1_ measured at 0–12 weeks could be evaluated by the quantitative MRI longitudinally. The repair of cartilage damage was also displayed by the 3D_WATSc and PDWI sequences, which was further confirmed by the gross observation and pathological findings.

However, there are some limitations in this study. First, SMNP incorporated into the hydrogel scaffold may interfere with magnetic field uniformity, making it difficult to evaluate neocartilage by other functional MR sequences. Second, the effects of SMNP concentration on hydrogel cross‐linking and growth factor release are unknown, which needs to be further investigated.

## 4 CONCLUSIONS

In summary, we designed and developed a multifunctional SMNP/KGN loaded hydrogel scaffold system for cartilage regeneration. The SMNP‐KGN/Gel possessed favorable mechanical property, suitable degradation rate, sustained KGN release, and sufficient MRI contrast enhancement. The multiparametric MRI can be used to monitor the degradation and functionalization of hydrogel scaffold noninvasively and longitudinally in vivo. This theranostic tissue engineering scaffold provides a practical strategy for imaging‐guided precision therapy for articular cartilage defect repair.

## 5 MATERIALS AND METHODS

### 5.1 Materials and reagents

Gelatin (type A, from porcine skin) was bought from Sigma–Aldrich (Shanghai, China). Hyaluronic acid (*M*_w_ = 10,000) was obtained from Meilunbio (Dalian, China). Methacrylate anhydride, dopamine hydrochloride, iron (III) chloride hexahydrate, 1‐ethyl‐3‐3‐di(methylaminopropyl)‐carbodiimide (EDC) and N‐hydroxysuccinimide (NHS) were acquired from Aladdin Co., Ltd. (Shanghai, China). Kartogenin (KGN) and acetone were obtained from Macklin Co., Ltd. (Shanghai, China). Dulbecco's modified Eagle's medium (DMEM), fetal bovine serum (FBS) and penicillin/streptomycin were purchased from Gibco (GIBCO). Counting Kit‐8 (CCK‐8) was from BestBio Bio‐Technology Co., Ltd. (Shanghai, China).

### 5.2 Preparation of the hydrogel

#### 5.2.1 Fabrication of the GelMA/HAMA/CNC Hydrogel

GelMA (100 mg), HAMA (50 mg) and different content of CNC (0 mg, 10 mg and 20 mg) was dissolve in 1 ml of PBS buffer at 40°C and stirred homogeneously. The hydrogels were named Gel‐0‐CNC, Gel‐1‐CNC, and Gel‐2‐CNC, 0, 1, and 2 represent the mass fraction of CNC. The photo‐initiator LAP was added into the above solution, and then the hydrogels were formed by irradiated 30 s under the blue light (405 nm). The Gel‐1‐CNC hydrogel was named as Gel and finally chosen to incorporate with the SMNP and SMNP‐KGN nanoparticles.

#### 5.2.2 Preparation of SMNP

The synthesis method of SMNP was followed by the reported method.^2^ Briefly, dopamine hydrochloride (45 mg) and 6.2 mg of FeCl_3_·6H_2_O were fully dissolved in 130 ml of deionized water under stirring at 25°C for 1 h. Next, 450 mg Tris (2‐amino‐2‐hydroxymethylpropane‐1,3‐diol) was dissolved in 20 ml deionized water, then quickly injected into the established solution and reacted for 2 h. Finally, the SMNP was separated by centrifugation and washed three times with deionized water.

#### 5.2.3 Preparation of SMNP‐KGN

Hydrophobic KGN powder (10 mg) was dissolved in 10 ml solution (acetone: H_2_O = 1:9) with EDC/NHS (5% w/w) for carboxyl activation. Next, 10 mg SMNP was put into the above KGN solution. We obtained a homogeneous mixture by sonicating for 30 min. Next, the solution was shaken (120 r/min) at 37°C for 24 h in a shaker. The precipitated particles were collected by centrifuging (8000 r/min), and then frozen at −20°C. Finally, SMNP‐KGN NPs were obtained under lyophilization (−80°C).

#### 5.2.4 Preparation of SMNP‐KGN/Gel

The preparation of SMNP/Gel and SMNP‐KGN/Gel hydrogels were shown as follow: 10 mg of SMNP were dissolved in 10 ml of PBS buffer and ultrasonicated for 10 min. Next, 1000 mg of GelMA, 500 mg of HAMA, and 100 mg CNC were put into the above solution and stirred for 30 min. After 0.1% (w/v) photo‐initiator LAP was added, the SMNP/Gel hydrogel was formed by irradiated 30 s under 405 nm‐blue light. The SMNP‐KGN/Gel hydrogel was obtained following the same procedures.

### 5.3 Characterization of the hydrogel

Fourier transform infrared spectroscopy (FTIR, Bruker, VERTEX 70) was used to characterize the chemical constitution of gelatin, GelMA, MA, HA, HAMA. The background was KBr tablet and the scan ranged from 400 to 4000 cm^−1^, with 4 cm^−1^ resolution. Nuclear magnetic resonance spectrometer (Bruker, ASCENDTM 600) was applied for nuclear magnetic resonance (^1^H NMR) spectra measurements. Scanning electron microscope (SEM, Philips, XL‐30) was used to observe the morphology of the lyophilized hydrogels.

### 5.4 Mechanical properties

The rheological properties of GelMA/HAMA/CNC hydrogels (height = 2 mm; diameter = 15 mm) were recorded over 0.1–10 Hz in a constant strain mode using a rotational rheometer (Kinexus pro, UK). For the mechanical properties test, Gel‐1‐CNC hydrogels (height = 6 mm; diameter = 11 mm) were prepared.

### 5.5 In vitro degradation behavior

The Gel‐1‐CNC hydrogels were placed in PBS (pH = 5.5 or 7.4) with or without 0.02 U/mL collagenase to evaluate the degradation behavior at 37°C, respectively. The degradation ratio was calculated according to the following formula:

$$\text{degradation ratio}=\left(W_{D}-W_{d}\right)/W_{D}\times 100\%.$$

In this equation, the *W*_*D*_ represents the initial weight of the hydrogels, and *W*_*d*_ is the weight after degradation at predetermined time points.

### 5.6 In vitro MRI methods for *T*_1_ characterization

The 1/*T*_1_ values of the FePN were measured on a Philips Achieva 3.0T MRI system at 37°C. Different concentrations of KGN‐SMNP hydrogels were collected in 5 ml Eppendorf tubes before MRI. The concentrations of Fe^3+^ varied from 0.05 to 0.43 mM acquired from ICP. All the samples were imaged under the *T*_1_ WI (TR/TE = 500/8.2 ms) and the *T*_1_ mapping (TR/TE = 100–1500/10.6 ms). Other acquisition parameters include: slice thickness = 3 mm, slice spacing = 0.5 mm, FOV = 100 × 100 mm, matrix = 256 × 256. The *T*_1_ signal values were measured by defining the region of interest (ROI) of each tube. Relaxivity (*r*_1_) was analyzed via the fitting curve of the 1/*T*_1_ (s^−1^) relaxation times versus the Fe concentrations (mM).

### 5.7 Bioactivity of the hydrogels

The cytotoxicity of the prepared SMNP/Gel with different SMNP concentrations (0–0.5 mg/mL) was evaluated with a Cell Counting Kit‐8 (CCK‐8) on 24 h and 48 h after seeding. Cell proliferation of the hydrogels (Gel, SMNP/Gel, and SMNP‐KGN/Gel) was evaluated by the CCK‐8 assay after incubation for 1, 4, and 7 days. On day 7, BMSCs were labeled with a live/dead cell staining kit and observed under the fluorescence microscope. Moreover, the effect of KGN on the migration of BMSCs was studied by the cell scratch wound experiment. The migration of BMSCs in each well with or without KGN was observed and recorded after 12 h and 24 h of incubation. The quantitative real‐time PCR was used for cartilage‐specific gene (COLII, Aggrecan, SOX9, and COLI) expression was analysis (Supplementary materials).

### 5.8 MRI evaluation of cartilage defect repair in vivo

All of the animal experimental protocols were approved by Institutional Animal Care and Use committee of Nanfang Hospital, Southern Medical University. The sample size of experimental animals was estimated through the degree of freedom (*E*) of analysis of variance. Generally, 2–2.5 kg male rabbits were randomly divided into 3 groups (4 knees per group): Gel, SMNP/Gel, and SMNP‐KGN/Gel. Then osteochondral defects (diameter = 4 mm, depth = 3 mm) were made on the center of the trochlear groove by a drill. The prepared hydrogel was injected and irradiated with blue light for 30 s to gelatinize.

All animals underwent MRI after surgery at 3, 6, 9 and 12 weeks. The 3D‐WATSc sequence acquisition parameters: TR = 12 ms, TE = 4.8 ms, matrix size = 100 × 100, FOV = 4 cm × 4 cm, slices = 78, flip angle = 15° and in‐plane resolution = 12 cm × 12 cm; PDWI acquisition parameters: TR = 2000 ms, TE = 36 ms, matrix size = 150 × 150, FOV = 6 cm × 6 cm, slice thickness = 1 mm, flip angle = 90° and in‐plane resolution = 13 cm × 13 cm. The longitudinal relaxation of *T*_1_ was measured using the *T*_1_ mapping sequences: TR = 2000 ms, TE = 20 ms, IR = 100, 200, 300, 400, 500, 600, 700, 800, 900, 1000 ms.

### 5.9 Histological evaluation of repaired cartilage

The cartilage repair was evaluated by gross images of the femur condyles collected at week 6 and 12 after MRI examination. The International Cartilage Repair Society (ICRS) macroscopic score standard was used (Table S2) to assess the degree of defect regeneration. The harvested cartilages were fixed by 4% paraformaldehyde and then decalcified with 10% ethylenediaminetetraacetic acid for 3 months. After embedding in paraffin, H&E, Safranin O, Toluidine blue, and Prussian blue staining of the samples sections (5 μm) were conducted to evaluate the histology.

### 5.10 Statistical analyses

The data are expressed as the mean ± SD (*n* = 3). All statistical computations were exhibited by SPSS software (ver. 20.0; SPSS Inc., Chicago, IL). The homogeneity of variance was analyzed with *F*‐test. Differences in different groups were analyzed with one‐way analysis of variance (ANOVA) with Bonferroni as post‐hoc analyses for multiple comparisons. Repeated measurement ANOVA was used to evaluate differences among different time intervals for in vivo MRI experiments. Significant differences were presented as *P < 0.05, **P < 0.01, or ***P < 0.001.

## AUTHOR CONTRIBUTIONS

**Chuyao Chen:** Conceptualization (supporting); data curation (equal); investigation (lead); methodology (equal); writing – original draft (lead). **Shaoshan Huang:** Data curation (supporting); formal analysis (supporting); investigation (supporting); visualization (supporting); writing – original draft (supporting). **Zelong Chen:** Conceptualization (supporting); data curation (supporting); investigation (supporting); methodology (supporting); writing – review and editing (supporting). **Qin Liu:** Formal analysis (supporting); investigation (supporting); resources (supporting); writing – review and editing (supporting). **Yu Cai:** Conceptualization (supporting); formal analysis (supporting); methodology (supporting); writing – review and editing (supporting). **Yingjie Mei:** Conceptualization (supporting); data curation (supporting); formal analysis (supporting); writing – review and editing (supporting). **Yikai Xu:** Conceptualization (equal); funding acquisition (lead); project administration (equal); resources (equal); supervision (equal); writing – review and editing (equal). **Rui Guo:** Conceptualization (lead); funding acquisition (supporting); methodology (lead); supervision (equal); writing – review and editing (equal). **Chenggong Yan:** Conceptualization (lead); formal analysis (supporting); funding acquisition (lead); resources (equal); supervision (lead); writing – review and editing (lead).

## CONFLICT OF INTEREST

The authors have no conflicts of interest to declare.

### PEER REVIEW

The peer review history for this article is available at https://publons.com/publon/10.1002/btm2.10364.

## Supporting information


**Data S1** Supporting Information

## Data Availability

The data that support the findings of this study are available from the corresponding author upon reasonable request.
