# Supplementary material for: Kartogenin (KGN)/synthetic melanin nanoparticles (SMNP) loaded theranostic hydrogel scaffold system for multiparametric magnetic resonance imaging guided cartilage regeneration
Source: Bioeng Transl Med. 2022 Jun 29;8(1):e10364. doi: 10.1002/btm2.10364 (PMC9842022; doi:10.1002/btm2.10364)
Supplement: Supplementary file 1 — Data S1 Supporting Information [file BTM2-8-e10364-s001.docx]

**Supporting Information**

**Kartogenin** **(KGN)/synthetic melanin nanoparticles (SMNP) loaded theranostic hydrogel for multiparametric magnetic resonance imaging guided cartilage regeneration**

Chuyao Chen ^1,#^, Shaoshan Huang ^2,#^, Zelong Chen ^1^, Qin Liu ^1^, Yu Cai ^3^, Yingjie Mei ^4^, Yikai Xu ^1,^ *, Rui Guo ^2,^ *, and Chenggong Yan ^1,^ *

^1^ *Department of Medical Imaging Center, Nanfang Hospital, Southern Medical University, Guangzhou 510515, China*

^2^ *Key Laboratory of Biomaterials of Guangdong Higher Education Institutes, Guangdong Provincial Engineering and Technological Research Centre for Drug Carrier Development, Department of Biomedical Engineering, Jinan University, Guangzhou 510632, China*

*^3^ Clinical Research Centre, Zhujiang Hospital, Southern Medical University, Guangzhou, Guangdong, China; Centre of Orthopedics, Zhujiang Hospital, Southern Medical University, Guangzhou, Guangdong, China.*

*^4^ School of Biomedical Engineering, Southern Medical University, Guangzhou 510515, China*

# These authors contributed equally to this work.

**Supplementary materials and methods**

**1. Preparation of GelMA**

The gelatin (0.5 g) was dissolved in [deionized](javascript:;) [water](javascript:;) at 50 ℃ under magnetic stirring at 500 rpm. Then 0.4 mL of methacrylate anhydride was added dropwise and vigorously stirred at 50 ℃. The solution was dialyzed (MWCO = 4-8 kDa) in deionized water for 48 h at 40 ℃ to remove excess methacrylate anhydride and additional by-products. The resulting GelMA solution was frozen at -80 ℃ overnight and then lyophilized at -80 ℃ to obtain the final product.

**2. Preparation of HAMA**

The HAMA was synthesized according to the previous reports^1^. In brief, 0.5 g of HA was dissolved in [deionized](javascript:;) [water](javascript:;) at 4 ℃ under magnetic stirring at 500 rpm. Next, 0.4 mL of MA was dropped into the HA solution. Then 5 M NaOH was added to maintain the pH of the solution between 8 and 9 and kept at 4 °C under continuous stirring for 24 h. Subsequently, to remove excess methacrylate anhydride and additional by-products, the solution was dialyzed (MWCO = 4-8 kDa) in deionized water for 48 h. The resulting HAMA solution was frozen at -80 ℃ overnight and then lyophilized at -80 ℃ to obtain the final product.

**3. Characterization of the nanoparticles**

The diameter of SMNP and SMNP-KGN were characterized by transmission electron microscopy (TEM) scanner (JEM 2100F, JEOL, Japan) and nano laser particle size analyzer (Malvern, Zetasizer Nano ZS). The chemical constitution of SMNP, KGN and SMNP-KGN were characterized by Fourier transform infrared spectroscopy (FTIR, Bruker, VERTEX 70). The amount of KGN conjugated with SMNP was quantified using ultraviolet (UV) spectrophotometry. The conjugation efficiency of the KGN was calculated as follows:

Conjugation efficiency (%) = (KGN_before_–KGN_after_)/KGN_before_ × 100%

**4. *In vitro* drug release of KGN**

The drug-release process was evaluated as follows: the hydrogels were immersed in 3 mL PBS (pH 7.4). At specified time intervals, 1 mL incubation solution was taken, and the residual solution was supplemented with 1 mL PBS. The amount of KGN released was determined by UV spectrophotometry (KGN, the absorbance at 325 nm).

**5. Extraction and culture of bone-marrow mesenchymal stem cells (BMSCs)**

All animal experiments were approved by the Medical Ethics Committee on Animal Care of Southern Medical University. BMSCs were isolated from the marrow of 4-weeks-old New Zealand white rabbits. The marrow was removed from the bone with sterile surgical instruments and centrifuged at 400×g for 20 min. After removing the supernatant, a medium (American Gibco) containing 10% fetal bovine serum (FBS, Gibco, USA) was re-suspended and inoculated in culture bottles. Nonadherent cells were removed after incubation for 72 h. At 80%–90% confluence, the BMSCs were serially passage. In this study, 3-5 generation BMSCs were used in further experiments.

**6. Cytotoxicity**

Hydrogels containing different concentrations of SMNP (0-0.5 mg/ml) were incubated in medium (10% FBS) for 24 h, followed by leachate collection. The cytotoxicity of the prepared hydrogels was evaluated with a Cell Counting Kit-8 (CCK-8) at 450 nm on 24 h and 48 h after seeding. BMSCs were inoculated in 96-well plates at a concentration of 3×10^4^ cells/mL (100 μL per well, n=5). After 24 h, the medium was replaced with hydrogel leachate. Next, the leachate was discarded after 24 h or 48 h, and the medium containing 10% CCK-8 was added. After incubation for 1 h, the absorbance was measured at the wavelength of 450 nm with a microplate reader.

**7. Cell proliferation**

Hydrogels (Gel, SMNP/Gel, and SMNP-KGN/Gel) were incubated in a culture medium for 24 h to collect leachate. BMSCs were seeded on 48-well plates with 1.5×10^3^ cells per well. Different hydrogel leachates were added to the wells when BMSCs adhered. After incubation for 1, 4, and 7 days, cell proliferation was measured by the Cell Counting Kit-8 (CCK-8) assay. On day 7, BMSCs were labeled with a live/dead cell staining kit and observed under the fluorescence microscope.

**8. Cell migration**

The effect of KGN on the migration of BMSCs was studied by the cell scratch wound experiment. The BMSCs were inoculated in 6-well plates until fusion and scraped through the center with a 1 mL pipette, forming a cross in each well. Then, hydrogel leachates with or without KGN were added. The migration of BMSCs in each well was observed and recorded after 12 h and 24 h of incubation.

**9. Cartilage-specific gene expression analysis**

BMSCs were seeded into 6-well plates (5×10^5^/mL) and cultured in hydrogel extract for 14 days. It was then digested with 0.25% trypsin solution and collected after brief centrifugation. Total cellular RNA was isolated from the collected cells with Trizol reagent and then quantified using the 260/280 absorbance ratio on the nanodrop ND1000 spectrophotometer and reverse transcribed into cDNA using PrimeScript reverse transcriptional master mixture. The expression level of related genes was detected by adding cDNA and the same amount of forward and reverse primers S-11 into the SYBR reaction mixture. The relative expression levels of each chondrogenic gene were calculated using the 2-△△△Ct method, and GAPDH was used as an internal control. The specific primers are listed in Table S1.

**10.Cartilage defect model**

All animal procedures were approved by Institutional Animal Care and Use committee of Nanfang Hospital, Southern Medical University. Generally, 2- 2.5 kg male rabbits were randomly divided into 3 groups (4 knees per group): Gel, SMNP/Gel, and SMNP-KGN/Gel. After removing hair on the knees, the patellar and medial parapatellar arthrotomy was laterally dislocated. Then osteochondral defects with 4 mm in diameter, 3 mm in depth were created on the center of the trochlear groove using a drill. The prepared hydrogel was injected and irradiated with blue light for 30 s to gelatinize. The defects treated with PBS were used as a control. The rabbits were injected with penicillin every 3 days to prevent infection and were allowed to move freely after waking.

**11. Gross observation of repaired cartilage**

After MRI examination at 6 and 12 weeks, gross images of the harvested femur condyles were taken to assess the cartilage regeneration. The International Cartilage Repair Society (ICRS) macroscopic score standard was used (Table S2), which evaluates the degree of defect repair, integration to the border zone, macroscopic appearance, and an overall repair assessment.

**Table S1** Primers for rt-qPCR.

| **Genes** | **Forward primer (5'->3')** | **Reverse primer (5'->3')** |
| --- | --- | --- |
| Collagen I | GAGATGAATGCAACGGCAAAA | CACCCCAGAAACAGACGACAA |
| Collagen II | ACAGTCTTGCCCCACTTACCG | GCTCCCAGAACATCACCTACC |
| Aggrecan | CTCCAGAAACCAGGTCAGGGA | GGTCCACCATTCGGCATAACT |
| Sox9 | GCTGTTTCTTCGGTCACTTTG | CAGCCTCTACTCCACCTTCAC |
| GAPDH | TGGGATGGAAACTGTGAAGAG | TTTGGCTACAGCAACAGGGTG |

**Table S2** ICRS macroscopic evaluation of cartilage repair.

| **Characteristic** | **Grading** | **Score** |
| --- | --- | --- |
| **Degree of defect repair** | In level with surrounding cartilage | 4 |
|  | 75% repair of defect depth | 3 |
|  | 50% repair of defect depth | 2 |
|  | 25% repair of defect depth | 1 |
|  | 0% repair of defect depth | 0 |
| **Integration to the border zone** | Complete integration with surrounding cartilage | 4 |
|  | Demarcating border <1mm | 3 |
|  | 3/4 of graft integrated with the surrounding | 2 |
|  | With a notable border >1mm width and 1/2 of graft integrated with the surrounding | 1 |
|  | From no contact to 1/4 of graft integrated with surrounding cartilage | 0 |
| **Macroscopic appearance** | Intact smooth surface | 4 |
|  | Fibrillated surface | 3 |
|  | Small, scattered fissures or cracks | 2 |
|  | Several, small or few but large fissures | 1 |
|  | Total degeneration of the grafted area | 0 |
| **Overall repair assessment** | Grade I: normal | 12 |
|  | Grade II: nearly normal | 11-8 |
|  | Grade III: abnormal | 7-4 |
|  | Grade IV: severely abnormal | 3-1 |

**Supplementary Figures**


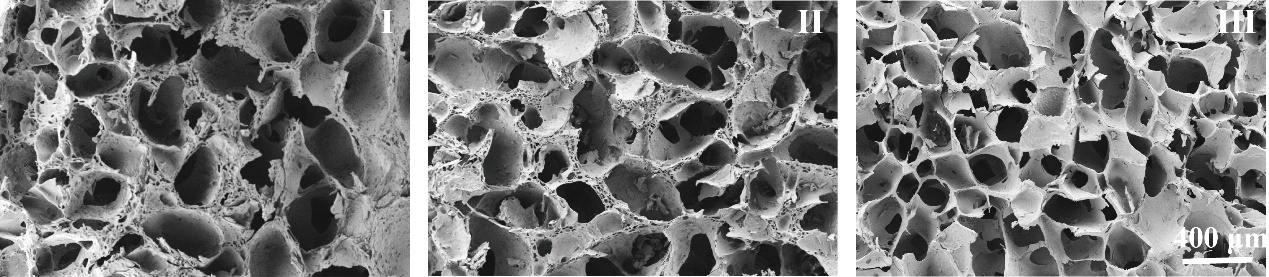
**FIGURE S1**. SEM images of the hydrogel. (I. 10% GelMA + 5% HAMA+0% CNC, II. 10 % GelMA +5% HAMA+1% CNC, and III. 10 % GelMA + 5% HAMA+2% CNC; scale bar: 400 µm.)

**FIGURE S2** *In vitro* degradation analysis of hydrogels immersed in PBS solution (pH=5.5) with or without collagenase at 37°C.

**

**

**FIGURE S3** The size distribution of SMNP measured by DLS.

**

FIGURE S4** The standard curve of KGN concentration.


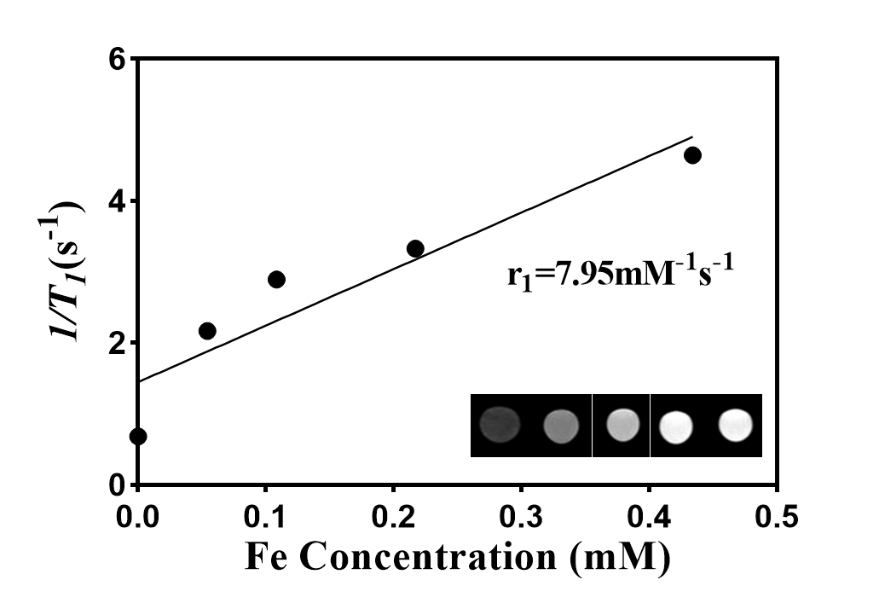


**FIGURE S5** The *r*_1_ value and MR images of SMNP-KGN /Gel in the concentration of 0, 0.25, 0.5, 1, 1.5 mg/ml, respectively.

**REFERENCES**

1 SHI D, XU X, YE Y, et al. Photo-Cross-Linked Scaffold with Kartogenin-Encapsulated Nanoparticles for Cartilage Regeneration [J]. ACS Nano, 2016, 10(1): 1292-9.

2 HAN L, WANG M, LI P, et al. Mussel-Inspired Tissue-Adhesive Hydrogel Based on the Polydopamine-Chondroitin Sulfate Complex for Growth-Factor-Free Cartilage Regeneration [J]. ACS Appl Mater Interfaces, 2018, 10(33): 28015-26.
